# Lack of WWC2 Protein Leads to Aberrant Angiogenesis in Postnatal Mice

**DOI:** 10.3390/ijms22105321

**Published:** 2021-05-18

**Authors:** Viktoria Constanze Brücher, Charlotte Egbring, Tanja Plagemann, Pavel I. Nedvetsky, Verena Höffken, Hermann Pavenstädt, Nicole Eter, Joachim Kremerskothen, Peter Heiduschka

**Affiliations:** 1 Department of Ophthalmology, University of Münster Medical School, 48149 Münster, Germany; viktoria.bruecher@ukmuenster.de (V.C.B.); charlotte.egbring@yahoo.de (C.E.); tanja.plagemann@ukmuenster.de (T.P.); nicole.eter@ukmuenster.de (N.E.); 2 Department of Nephrology, Internal Medicine D, Hypertension and Rheumatology, University of Münster Medical School, 48149 Münster, Germany; nedvetsky@uni-muenster.de (P.I.N.); Hermann.Pavenstaedt@ukmuenster.de (H.P.); Joachim.Kremerskothen@uni-muenster.de (J.K.); 3 Medical Cell Biology, Medical Clinic D, University of Münster Medical School, 48149 Münster, Germany; verena.hoeffken@ukmuenster.de

**Keywords:** WWC protein, angiogenesis, Hippo signalling pathway, hypersprouting, endothelial cell specific knock-out

## Abstract

The WWC protein family is an upstream regulator of the Hippo signalling pathway that is involved in many cellular processes. We examined the effect of an endothelium-specific WWC1 and/or WWC2 knock-out on ocular angiogenesis. Knock-outs were induced in C57BL/6 mice at the age of one day (P1) and evaluated at P6 (postnatal mice) or induced at the age of five weeks and evaluated at three months of age (adult mice). We analysed morphology of retinal vasculature in retinal flat mounts. In addition, in vivo imaging and functional testing by electroretinography were performed in adult mice. Adult WWC1/2 double knock-out mice differed neither functionally nor morphologically from the control group. In contrast, the retinas of the postnatal WWC knock-out mice showed a hyperproliferative phenotype with significantly enlarged areas of sprouting angiogenesis and a higher number of tip cells. The branching and end points in the peripheral plexus were significantly increased compared to the control group. The deletion of the WWC2 gene was decisive for these effects; while knocking out WWC1 showed no significant differences. The results hint strongly that WWC2 is an essential regulator of ocular angiogenesis in mice. As an activator of the Hippo signalling pathway, it prevents excessive proliferation during physiological angiogenesis. In adult animals, WWC proteins do not seem to be important for the maintenance of the mature vascular plexus.

## 1. Introduction

The Hippo signalling pathway is involved in a wide range of processes within the human body, such as regulation of organ size, cell proliferation, and tumorigenesis [1,2,3]. The core of Hippo signalling pathway in mammalian cells consists of two protein kinases, MST1/2 and LATS1/2, and a transcription factor YAP (or its homologue TAZ). MST1/2-dependent phosphorylation leads to activation of LATS1/2, which culminates in phosphorylation YAP and TAZ preventing their import into the nucleus and terminating expression of YAP/TAZ-dependent target genes. Numerous proteins involved in regulation of cell growth, polarity, and homeostasis regulate activity of Hippo signalling pathways (for recent reviews see [4,5]).

Within the past decades, more and more proteins involved in fine-tuning of Hippo pathways have been discovered, among them the WWC protein family (for WW-and-C2-domain-containing-protein) [6], which is an upstream regulator of the Hippo signalling pathway. The member WWC1 was identified to be involved in synaptic signalling and also implicated in both higher brain functions and cell polarity or rather migration of, for instance, podocytes [7,8,9,10]. Current studies have examined its role in different cancer types, such as breast cancer, gastric cancer, and lung adenocarcinoma [11,12,13]. Besides WWC1, the genomes of mammals, unlike invertebrates, encode a whole WWC protein family, which comprises the highly similar WWC2 and WWC3 [14].

WWC proteins have in common the binding site for aPKC, a class III PDZ-interaction motif (ADDV) and homologous phosphorylation motifs [14]. The phospho-regulation is required for proper cell proliferation and also modulates the migratory activity [15]. Another similarity between the WWC proteins is the ability to form hetero- or oligomeric complexes and associate with dynein light chain 1 (DLC1) and atypical protein kinase C (aPKC) [14,16]. The very high expression in the lung is also a common feature of the WWC proteins. However, WWC2 was detected by qRT-PCR mainly in the testis, whereas ovary tissue displayed increased levels of WWC3 [14].

WWC1 was shown to be an upstream component of the Hippo signalling pathway [17,18,19,20]. In *Drosophila*, it is localised to the apical domain of epithelial cells. It forms a complex with the FERM domain proteins Merlin (Mer) and Expanded (Ex), regulating the Hippo kinase cascade via direct binding to Hpo and Sal [17,18,19]. In human cells, the association between WWC1 and its orthologues seems to be conserved [17,20]. It impacts the phosphorylation status of the downstream effectors YAP/TAZ, the most crucial step in Hippo signalling [21]. An overexpression enhances and a depletion reduces YAP phosphorylation, so that its nuclear import and therefore transcriptional activity is either prevented or not [14,20]. From *Drosophila* to human cells, this regulation is additionally mediated through interaction with protein tyrosine phosphatase, non-receptor type 14 (PTPN14), and angiomotin family proteins (AMOT) [22].

It has been shown that the downstream effector of Hippo signalling pathway, YAP, regulates retinal cell differentiation by promoting proliferation while inhibiting cell-cycle exit [23,24], indicating a decisive role in ocular development and disease.

Formation of a functional blood vessel network is essential for survival and function of any organs. Most of the blood vessels are formed during development by a process called angiogenesis, formation of new blood vessels from pre-existing ones. Angiogenesis can be investigated with the aid of several in vivo model systems, of which the mouse retina offers numerous advantages [25,26,27]. While retinal angiogenesis is widely studied as a proxy for angiogenic processes taking place in other organs, it is an integral part of eye development and essential for vision.

Recently, a role of transcription factors YAP/TAZ and several of their upstream regulators in angiogenesis have been demonstrated. Endothelial cell (EC)-specific knock-out of the upstream regulator LATS resulted in a hypersprouting phenotype in the mouse retina model [28]. Likewise, Sakabe et al. took a similar approach to delete the members of the Hippo pathway YAP and TAZ in ECs in a temporally regulated manner, leading to a severe vascular phenotype with prominently impaired retinal vessel sprouting, decreased vascular area, and reduced number of vascular branches [29]. Proper regulation of YAP/TAZ activity might be also important for maintenance of the blood vessels, since activity of YAP and TAZ is high in case of disturbed blood flow, and YAP/TAZ activities are suppressed in athero-protective uniform laminar shear stress. Altogether, these data indicate that an inhibition of YAP/TAZ could be a promising therapeutic strategy for atherosclerosis [30,31], pulmonary hypertension [32], and cancer [33].

While the role of YAP/TAZ during angiogenesis becomes more and more clear, the interplay between the upstream components regulating these transcription factors is still elusive. For instance, it is unclear whether the same or different upstream components regulate YAP/TAZ activity during development and adulthood. Equally unknown is whether there is a tissue-specific difference in composition of Hippo signalling pathway. Recently, we have demonstrated that WWC2 protein plays an important role during embryonic and postnatal angiogenesis [34]. In this study, we analysed whether WWC2 is only important for retinal angiogenesis during development or also required for vascular maintenance.

## 2. Results

### 2.1. Postnatal Animals

Pups (74 in total) were intragastrically injected with 50 µg tamoxifen three times, on postnatal days P1, P2, and P3. At P6, retinas were isolated and stained according to the protocol [35]. To evaluate retinal vasculature, 138 retinas were stained with isolectin B4. In addition, 68 retinas were stained with a specific antibody directed against the tip cell marker ESM1.

**Figure 1:**
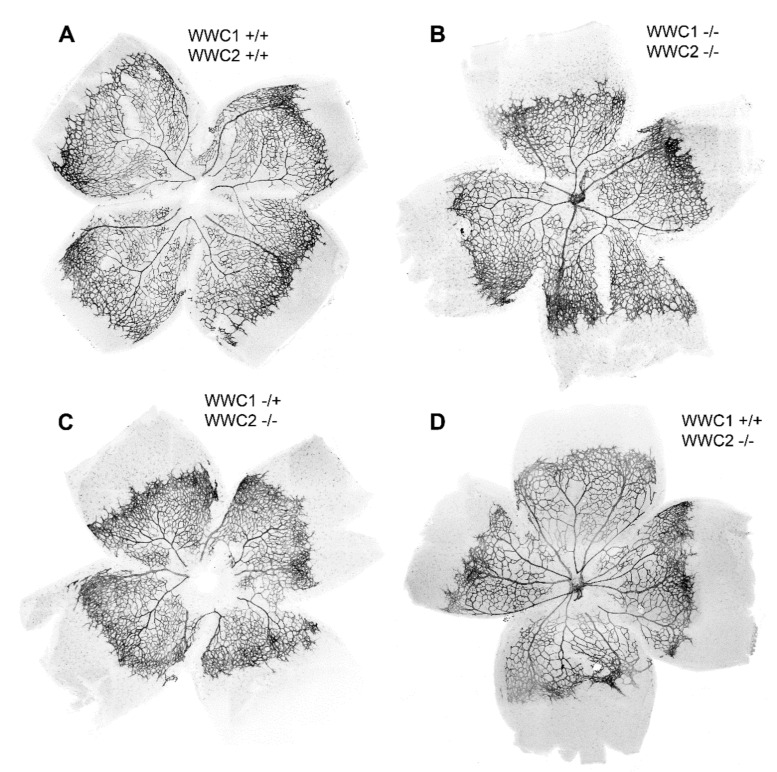
Retinal whole mounts from P6 mice with blood vessels labelled with isolectin B4. The control is shown in (**A**), and the three knock-out groups in (**B**–**D**). The latter did not express WWC2 and differed in their WWC1 expression as indicated. They exhibited a dense vascular network in the peripheral plexus, the so-called, “hypersprouting areas”, and a smaller portion of the retinal vascularisation.

**Figure 2:**
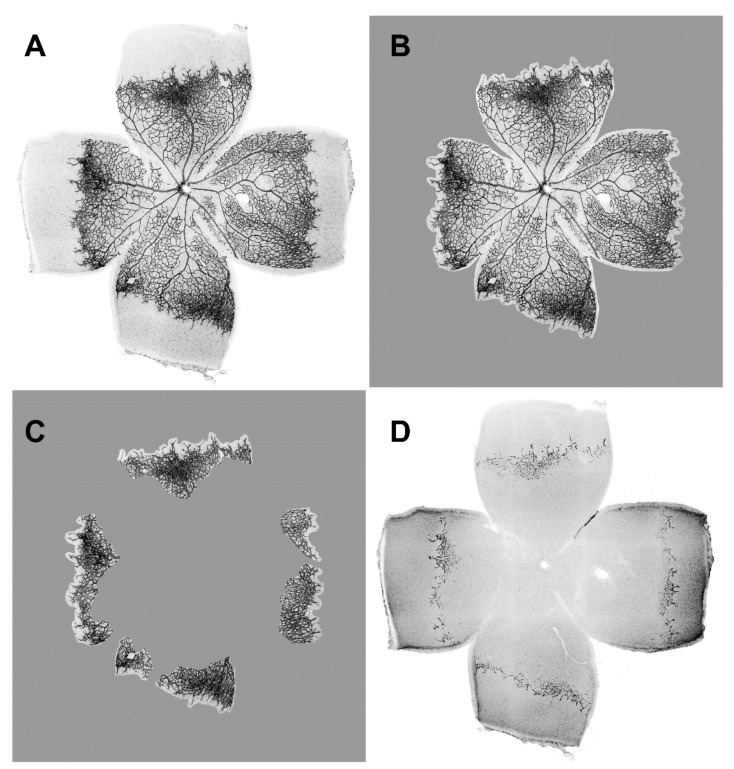
Example for evaluation of a retinal whole-mount. Digital image of a complete retinal whole-mount is shown in (**A**). In this example, size of total retinal area was determined to be 19,752,079 pixels. Size of vascularised area was 13,578,789 pixels (**B**), and size of hypersprouting area 4,607,985 pixels (**C**). According to this, sprouting had a 33.9% share of vascularised area. Staining against the endothelial cell-specific molecule 1 (ESM1) displays the tip cells which helped to determine the hypersprouting areas (**D**).

Typical examples of the appearance of retinal whole mounts of the four genotypes are shown in Figure 1. In the knock-out animals (WWC1^+/+^WWC2^−/−^, WWC1^−/+^WWC2^−/−^, and WWC1^−/−^WWC2^−/−^), areas of an enhanced density of blood vessels in the periphery, which were not seen to that extent in the control retinas, caught our eye. We called these areas of the peripheral plexus exhibiting a dense vascular network, “hypersprouting areas”. As a first step, we determined which portion of the total vascular plexus was occupied by these hypersprouting areas by a method shown in Figure 2.

**Figure 3:**
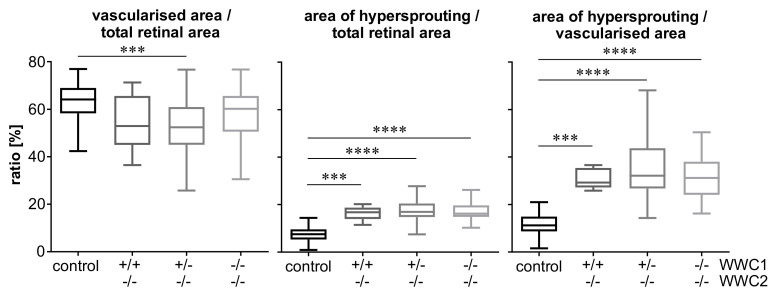
Results of evaluation of vascularised and hypersprouting areas as indicated in P6 retinal whole-mounts. In this and the following diagrams, data are presented of *n* = 42 control eyes, *n* = 8 WWC1^+/+^WWC2^−/−^ eyes, *n* = 56 WWC1^+/−^WWC2^−/−^ eyes, and *n* = 23 WWC1^−/−^WWC2^−/−^ eyes. Statistical significance of differences between the groups is denoted by asterisks: *** *p* < 0.001, **** *p* < 0.0001.

Evaluation of vascularised and hypersprouting areas revealed that a knock-out of the WWC2 gene leads to a slight decrease of vascularised area compared to total retinal area and to a clear and highly significant increase of hypersprouting area compared to both total retinal area and vascularised area (Figure 3). Extends of these effects were similar in the three WWC2 knock-out groups, independent of the expression status of the WWC1 gene, and indicating that WWC1 was not able to compensate for WWC2 loss and prevent vascular abnormalities.

**Figure 4:**
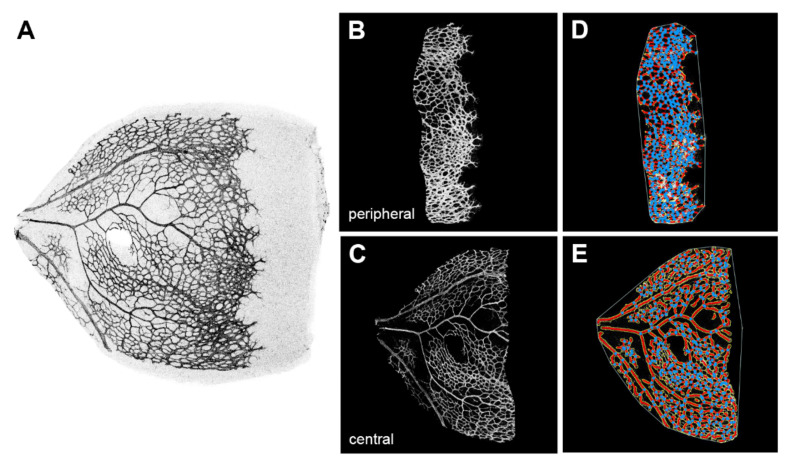
Example for the evaluation of a retinal leaf using the AngioTool software. The retinal leaf (**A**) was divided into a peripheral part (**B**) and a central part (**C**). Vessels (red) and junctions (blue dots) were detected by the software in the digital images (**D**,**E**).

For further analysis, we evaluated single leaves of the four-leaf clover-shaped whole mounts, and we divided vascularised areas of the leaves into a central area where remodelling of vasculature was already visible, and a peripheral area where remodelling was not accomplished. Morphometric parameters such as overall size of vascular network, vascular density, total and average vessel length, number of vessel endpoints, and lacunarity were determined separately for these peripheral parts and central parts. Examples of such a separation into a central part and a peripheral part as well as evaluation are shown in Figure 4.

**Figure 5:**
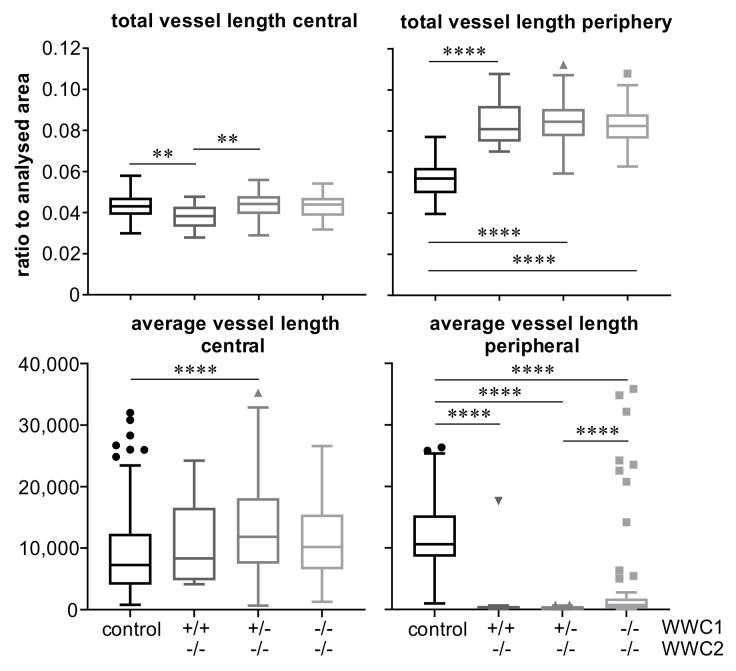
Diagrams showing values of total vessel lengths, normalised to the analysed area, and average vessel lengths between junction points as indicated. Circles, triangles and squares indicate outliers according to Tukey’s presentation of box plots. Statistical significance of differences between the groups is denoted by asterisks: ** *p* < 0.01, **** *p* < 0.0001.

Vessel lengths of central and peripheral parts were normalised to the analysed areas of the corresponding retinal parts. Moreover, average vessel lengths between two junction points were analysed. In central areas, we found a rather heterogeneous picture with slight differences between the groups, which reached statistical differences in some cases. In contrast, total vessel lengths and average vessel lengths differed in the same way and with a high significance in peripheral areas between the controls and all three knock-out groups (Figure 5).

**Figure 6:**
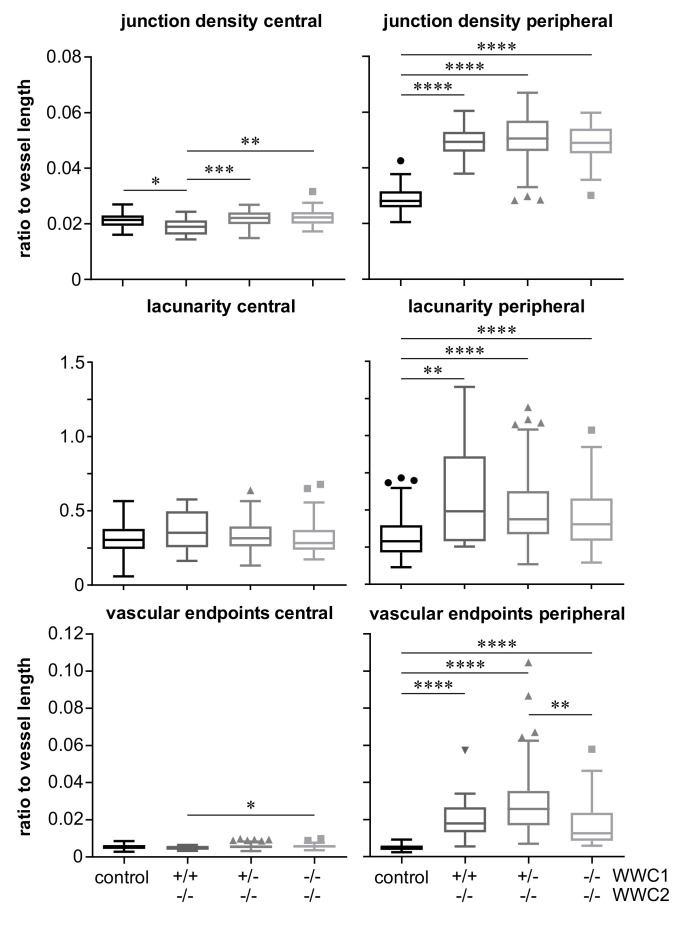
Diagrams showing values of junction density, lacunarity, and vascular endpoints in P6 retinas as indicated. Junction density and number of endpoints were normalised to the total vessel length in the analysed areas. Triangles and squares indicate outliers according to Tukey’s presentation of box plots. Statistical significance of differences between the groups is denoted by asterisks: * *p* < 0.05, ** *p* < 0.01, *** *p* < 0.001, **** *p* < 0.0001.

A similar picture is seen in the analysis of the further parameters’ junction density, lacunarity, and vascular endpoints (Figure 6). Again, there are minor differences between the groups in the central parts of the retina, which sometimes reach statistical significance, and clear differences with a high significance between the controls and all three knock-out groups.

### 2.2. Adult Animals

**Figure 7:**
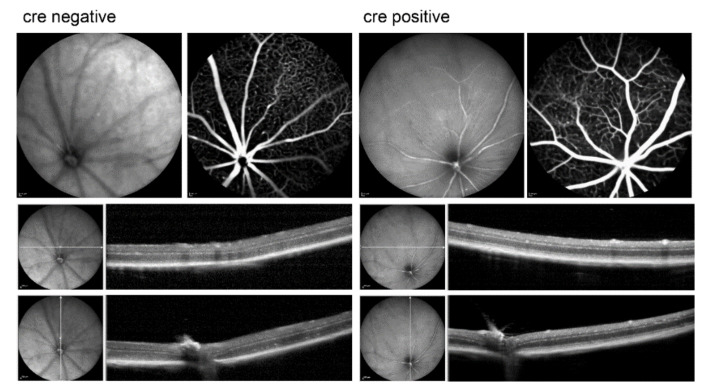
Examples of in vivo imaging of Cre-negative mice (controls) and Cre-positive mice (knock-outs) as indicated. On top, images are shown obtained by infrared scanning laser ophthalmoscopy (**left**) and fluorescein angiography (**right**). Below, optical coherence tomography images are shown, with both horizontal and vertical scans.

*17.* WWC1^−/−^WWC2^−/−^ mice were treated with tamoxifen at the age of five weeks and were inspected seven weeks later at the age of 12 weeks. Among the animals, we had six Cre-negative mice and 11 Cre-positive mice, constituting the two experimental groups’ controls and knock-outs, respectively. In vivo imaging by fluorescence angiography and optical coherence tomography did not reveal differences between the controls and the knock-out animals (Figure 7).

**Figure 8:**
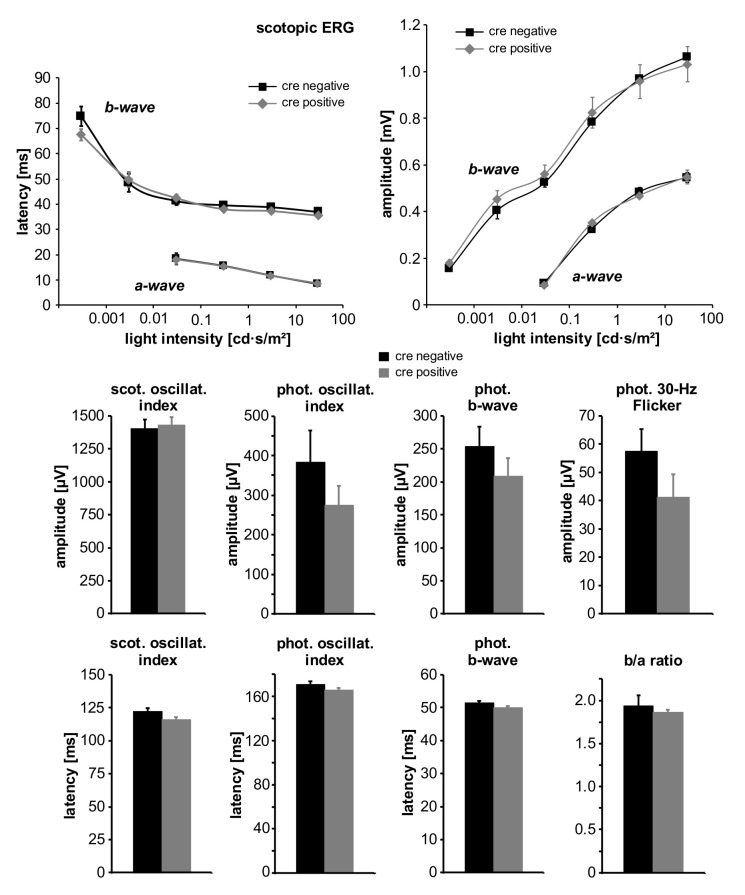
Values of different ERG parameters obtained in Cre-positive (knock-outs) and Cre-negative (controls) WWC1^−/−^WWC2^−/−^ mice. On top, latencies and amplitudes in scotopic ERG are shown depending on intensity of light stimulus. Below, amplitudes and latencies of scotopic and photopic oscillatory index, photopic b-wave, photopic 30-Hz Flicker ERG, and scotopic b/a ratio are shown as indicated.

Electroretinographic measurements were performed in seven Cre-positive mice (knock-outs) and three Cre-negative mice. Comparison of ERG parameters (latencies and amplitudes) did not reveal any differences between the two groups (Figure 8). There was a trend for photopic amplitudes, i.e., cone activity, to be smaller in the Cre-positive group compared to the Cre-negative group. Nevertheless, the difference was not statistically significant (Figure 8).

**Figure 9:**
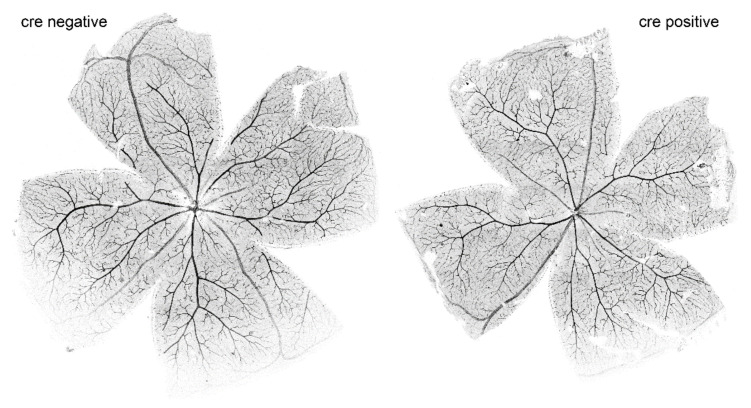
Retinal whole mounts from 12-week-old mice with blood vessels labelled with Isolectin B4.

After in vivo imaging and ERG measurements, retinas were isolated and blood vessels stained in the whole mounts. On first sight, we did not see any relevant differences between the retinas of Cre-negative and Cre-positive mice (Figure 9).

**Figure 10:**
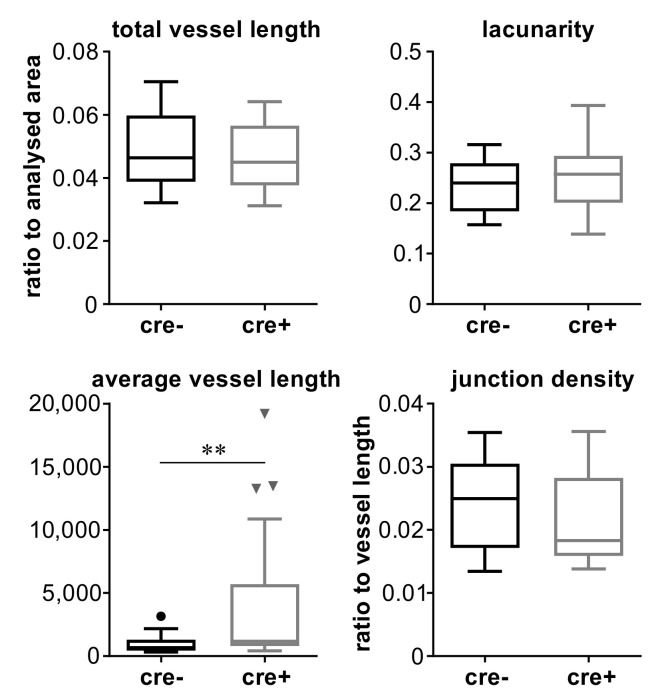
Diagrams showing values of total vessel length normalised to analysed area, lacunarity, average vessel length, and junction density normalised to total vessel length in 12-week-old retinas of Cre-positive mice (knock-outs) and Cre-negative mice (controls) as indicated. Triangles and circles indicate outliers according to Tukey’s presentation of box plots. Statistical significance of differences between the groups is denoted by asterisks: ** *p* < 0.01.

In order to check further properties of the vascular network, retinas of both groups were evaluated using the AngioTool software, similarly to the retinal whole mounts of P6 mice. Several parameters of the vascular network were determined (Figure 10). There were no differences between the two groups except for the average vessel length between two junction points.

## 3. Discussion

The main three findings of this study were that (1) in the perinatal mice, the loss of WWC proteins caused a vascular hypersprouting, measured by highly significant larger percentages of sprouting area, an increased amount of tip cells, higher junction density, longer total and shorter average vessel length, and more endpoints and lacunarity in comparison to the control group. (2) In adult mice, there were neither morphological nor functional differences between the WWC1/2 knock-out and the control group, demonstrating that the double knock-out does not cause retinal impairment in adulthood. (3) In addition, we identified WWC2 to be the crucial regulator of angiogenesis and WWC1 to not be important in this context.

### 3.1. Knock-Out in Perinatal Animals

In earlier studies, the phenotype of hypersprouting was observed upon experimental blocking of DLL4 or inhibition of Notch signalling [36,37,38]. An analogical phenotype has been reported by Kim et al. examining an endothelial cell specific LATS knock-out [28]. A similar observation has been made in the same year by Sakabe et al., who also generated an inducible endothelial cell-specific LATS knock-out and described a hyperplastic angiogenic network with an almost doubling of branching points and a significant increase of proliferation rate [29]. In the following year, Neto et al. generated a TAZ gain-of-function mice and obtained analogical outcomes [39]. The results of our study are in line with these findings, as LATS acts downstream of WWC in the Hippo pathway. Thus, they support the hypothesis that in case of loss of WWC, Hippo pathway is less activated and therefore YAP and TAZ less phosphorylated. As a consequence, YAP and TAZ can enter the nucleus and enhance the transcription of its target genes, resulting in increased proliferation and, referring to the retinal vasculature, hypersprouting. These transcriptional targets include activators of the integrin αVβ3 pathway CTGF and CYR61 [40,41,42], ANG-2 [43], DLC1 [44], and many other pro-proliferative and anti-apoptotic genes.

Remarkably, the three studies cited above also explored the corresponding counterpart by generating YAP/TAZ double knock-out mice [28,29,39]. As well as upon investigation of a YAP/TAZ knock-down by short interfering RNA [23], reduced vascular density, branching frequency, impaired sprouting, and an overall hyperpruned vascular network were observed. With regard to these findings, it would be challenging but particularly intriguing to explore in further studies whether a hyperactivation of WWC would reproduce similar results. In another study, Nedvetsky et al. identified inhibition of endothelial protein kinase A as a further cause for retinal hypersprouting [45]. Interestingly, they also compared 1-month-old wild-type and knock-out mice and observed no obvious differences, concluding that remodelling can correct the patterning defects. Another important approach to investigate consequences of a WWC knock-out will be to inject tamoxifen postnatally and thereupon evaluate retinal vasculature in adult mice at the age of 12 weeks.

This study reveals a highly dense, ESM1-positive, and increased amount of tip cells in the retinas of knock-out mice. As seen in the findings of Stenzel et al., the surrounding endothelial basement membrane limits tip cell formation [46]. To exclude the possibility that the observed phenotype is caused by a damage of the endothelial basement membrane, we stained the retinas for collagen IV, one of its components. Retinas of both control and knock-out group demonstrated the same staining pattern (not shown). Thus, it is unlikely that the WWC knock-out impairs the capacity of ECs to deposit basement membrane proteins. Another essential external factor for the vascular network formation could be astrocytes [47]. The staining with GFAP showed neither noticeable defects nor any differences in its expression pattern between the control and knock-out group (not shown). Therefore, we conclude that angiogenesis may not be impaired due to a disturbed interaction between ECs and astrocytes.

### 3.2. Knock-Out in Adult Animals

In 2017, Kim et al. deleted YAP/TAZ in ECs at adulthood [28]. The mice did not show abnormal phenotypes, indicating that YAP/TAZ is not required to maintain blood-brain barrier and blood-retinal barrier integrity. These results are in line with our findings, as we found that WWC, an upstream component of YAP/TAZ, was dispensable to maintain normal vasculature under physiological conditions. Therefore, neither a higher nor lower activity of endothelial YAP/TAZ impacts on vascular maintenance. In the last years, many new insights were gained regarding vessel maintenance, identifying it as a cooperation of many constituents of blood vessels such as ECs, but also the ECM and pericytes [48]. Since both different cell types and signalling pathways are involved in this complex process, we speculate that either the WWC proteins do not play a major role or their loss can be compensated. To get to the bottom of this assumption, one approach would be to investigate the adult mice a few days after their knock-out induction, thus at the age of approximately six weeks, and research whether they show abnormalities.

### 3.3. WWC2 Is the Decisive Factor for Vascular Development, While WWC1 Is Not Pivotal

As presented before, none of the three different postnatal knock-out groups expressed WWC2. They just differed in their expression level of WWC1, either exhibiting a homozygous or heterozygous knock-out or a wild-type gene expression. The quantitative results revealed highly significant larger percentages of hypersprouting in every knock-out group compared to the control group, whereas no significant differences among the knock-out groups were found (Figure 3). This demonstrates that the hypersprouting phenotype is due to the loss of WWC2, because otherwise differences between the knock-out groups should be visible. The same observation is made in the computational analysis of the peripheral parts of the retina as sketched in Figure 4. The ascertained higher junction density, longer total and shorter average vessel length, increased number of endpoints, and enhanced lacunarity could be observed in all three groups, with invariably very high statistical significances in comparison to the control group (Figure 5 and Figure 6). Our data point towards the hypothesis that WWC2 is the crucial member of the protein family in mice with regard to angiogenesis and WWC1 is in that respect not decisively important, although they share a total sequence identity of 49% [14].

The observation that absence of WWC1 does not modulate angiogenesis was not necessarily unexpected, as a functional redundancy between WWC1 and WWC2 was shown in other cell types. An induced loss of WWC1 leads to a mild cognitive phenotype without deficits in kidney or lung function [8,49]. Moreover, in hepatocytes, the genetic ablation can be completely compensated by only one representative of the WWC proteins [50]. Additionally, beyond that, the deletion of the WWC3 locus in the *M. musculus* lineage showed no phenotypic effects [51]. Even other members of the Hippo pathway, such as YAP/TAZ, were shown to regulate each other in a compensatory way [38]. These data suggest a genetic redundancy in the WWC family.

The mouse organogenesis cell atlas (MOCA) [52] displays a very high expression of the WWC2 gene in vascular ECs, which includes tip and stalk cells. In contrast, WWC1 is primary expressed in epithelial cells. Moreover, recent studies showed that a WWC2 deletion results in severe defects in angiogenesis [34]. These data confirm our findings about WWC1 playing a subordinate role in angiogenesis, as it is of secondary importance for the most essential cell type of blood vessels, the ECs. Furthermore, the MOCA explains the discrepancy between our findings and the studies mentioned above, as other cell types present completely different expression patterns.

Unfortunately, good antibodies against the downstream proteins LATS and YAP were not available. Therefore, we were not able to identify changes in these proteins after knock-out of the WWC genes, which is a limitation of the present work. Another limitation is that partition of the retinal flat-mounts into a peripheral hypersprouting part and a central normally vascularised part was performed by subjective judgement instead of an automated procedure. To minimise the subjective factor, three investigators (C.E., V.B., and P.H.) had to agree on each partition without knowing to which group the retinas belonged.

While WWC2 seems to be dispensable for the maintenance of the adult blood vessels, it is not clear yet whether endothelial WWC2 plays a role in regulation of Hippo-YAP signalling under pathological conditions. Downregulation of another negative regulator of YAP activity, LATS2, by microRNA miR-224-3p seems to play an important role in pathogenesis of retinoblastoma (Song et al., 2020 [53]). The authors proposed that inhibition of the Hippo-YAP signalling pathway may be a way to block progression of retinoblastoma, a life-threatening tumour in new-born children. Moreover, hypoxia-induced YAP activation was found to promote ocular neovascularisation in mouse models of choroidal neovascularisation and oxygen-induced retinopathy [54]. These findings emphasize the pathological significance of abnormal YAP activity. It is therefore plausible that downregulation of WWC2 or impairment of its function may also lead to overactivation of YAP under certain conditions. This might be especially relevant for endothelial cells, since WWC1 seems to be unable to compensate for WWC2 loss in these cells.

Taken together, we describe the previously unidentified, indispensable, and irreplaceable role of WWC2 in vascular angiogenesis. Since both ocular disease therapies, such as AMD and PDR, and beyond that, cancer treatment, use the protective and beneficial effects of anti-angiogenic therapies, the knowledge we gained about WWC2 as a crucial regulator is of great relevance and importance. Further research analysing its function in humans is needed, especially because the role of WWC3 still remains unexplored.

## 4. Materials and Methods

### 4.1. Animals and Induction of the Knock-Out

Animal experiments including all procedures of treatment with tamoxifen, in vivo imaging and electroretinography were allowed by the responsible state animal ethics committee of the ‘Landesamt für Natur, Umwelt und Verbraucherschutz’ (LANUV North Rhine-Westphalia, Recklinghausen, ref. no. 81-02.04.2018.A381). Date of approval; 28. January 2019.

Generation of mice with a floxed WWC2 gene (WXC2^fl/fl^) and mice harbouring floxed WWC2 and WWC1 genes (WWC1^fl/fl-^WWC2^fl/fl^) were described before [50]. WWC1^fl/fl-^WWC2^fl/fl^ animals were crossed to the Cadherin 5 (Cdh5)-CreERT2 mouse line (generous gift from R. Adams, Max-Planck-Institute for Molecular Biomedicine, Münster, Germany), enabling an inducible endothelial cell-specific activation of Cre expression [55]. To activate CreERT2 and hence induce the knock-out, the animals received tamoxifen on three consecutive days. Knock-outs were induced in the animals at two different ages, resulting in two experimental protocols. In the first protocol, postnatal pups were injected intragastrically with tamoxifen (Sigma-Aldrich, Munich, Germany; T5648) on days P1 to P3 (50 µL, 1 mg/mL, diluted in corn oil). Pups were killed on day P6, and retinas were isolated for further analysis. In the second protocol, tamoxifen was administered to five-week-old mice by an intraperitoneal injection (20 mg/mL in corn oil, 0.125 mg tamoxifen per gram body weight), and animals were killed at an age of 12 weeks to isolate the retina after inspecting retinal structure and function by in vivo imaging and electroretinography (ERG).

### 4.2. In Vivo Imaging

To visualise the retinal vasculature in adult mice and retinal structure, fluorescence angiography (FA) and optical coherence tomography (OCT) were applied using the device Spectralis (Heidelberg Engineering, Heidelberg, Germany), as described by Alex et al. [56]. Briefly, animals were anesthetised by an intraperitoneal injection of a mixture of 130 mg/kg ketamine (bela-pharm, Vechta, Germany) and 2.7 mg/kg xylazine (aniMedica, Senden-Bösensell, Germany). Pupils were dilated by one drop of tropicamide (Pharma Stulln, Stulln, Germany) and one drop of neosynephrine (Ursapharm, Saarbrücken, Germany). For FA, 100 µL of diluted sodium fluorescein were injected intraperitoneally, and images were taken by excitation at 488 nm. OCT images were taken as single scans and volume scans.

### 4.3. Electroretinography

ERG measurements were performed as described, e.g., in Heiduschka et al. [57], to compare retinal function in control and knock-out mice. Briefly, mice were anaesthetised and pupils dilated as described above. In addition, cornea was desensitised by a drop of proparacaine (Ursapharm, Saarbrücken, Germany). Measurement was performed using the RetiPort device by Roland Consult, Brandenburg, Germany. Animals were dark-adapted overnight and prepared for the measurement in dim red light. Gold ring electrodes were put onto the cornea as working electrodes, and a gold wire put into the mouth of the mice served as a counter electrode. Scotopic ERG was recorded by a series of light flashes of increasing intensity, ranging from 0.0003 to 30 cd∙s/m^2^. After a light adaptation period of 10 min at 25 cd/m^2^, photopic ERG was recorded at 100 cd∙s/m^2^.

### 4.4. Immunohistochemical Staining of Blood Vessels

Isolated eyes were fixed for 30 min in 4% paraformaldehyde (Carl Roth GmbH, Karlsruhe, Germany) dissolved in phosphate-buffered saline (PBS) and washed twice for five minutes in PBS. The eyeball was opened, and the retina separated, incised to obtain a four-leaf clover shape. For staining, the retina was incubated in blocking buffer (1% BSA, Sigma-Aldrich, Munich, Germany, #A7030, and 0.3% Triton X100, AppliChem, Darmstadt, Germany, #A4975, in PBS) for two hours at room temperature or at 4 °C overnight, washed with PBS, and incubated with biotinylated isolectin B4 (Vector Laboratories, Eching, Germany; B1205, 1:25) or the primary antibody against the endothelial cell-specific molecule 1 (ESM1) (R&D Systems, Minneapolis, USA; AF1999, 1:100) overnight at 4 °C. ESM1 is a marker particularly for tip cells in a developing vasculature. After repeated washing, the retina was incubated with streptavidin conjugated with Cy3 (SigmaAldrich, Munich, Germany; S6402, 1:200) or a secondary antibody conjugated with Alexa488 (Invitrogen Life technologies, Darmstadt, Germany; A-11055, 1:200). Stained retinas were mounted on glass slides and inspected by a fluorescent confocal microscope (Leica TCS SP8 HCS A, Leica Microsystems, Wetzlar, Germany).

### 4.5. Evaluation of Retinal Whole-Mount Staining

On digital images of the stained retinal flat mounts, areas of hypersprouting within vascularised areas were identified independently by three different observers (C.E., V.B., and P.H.). To determine sizes of total retinal area, vascularised areas, and areas of hypersprouting, they were surrounded manually by the lasso tool and the quick selection tool in Adobe Photoshop C5. The number of pixels given in the histogram window served as a measure for the sizes of the above-mentioned areas.

A more detailed analysis of the vascular network was performed using the freeware software “Angiotool” from the National Institute of Health National Cancer Institute, Gaithersburg, USA. To operate this software, fluorescent grey-scale images were sharpened and converted into a black-and-white bitmap using Adobe Photoshop (Adobe, San Jose, CA, USA). Moreover, images were split to allow separate analysis of areas with “normal” vascularisation and areas of hypersprouting. If no hypersprouting was visible, the vascularised area was split into two parts, a ratio similar to the cases with hypersprouting. Using the Angiotool software, overall size of vascular network, total and average vessel length, number of branching points, vascular density, and lacunarity were determined [58].

### 4.6. Statistical Anazlysis

For the statistical analysis, Prism 8 (GraphPad Software, Inc., La Jolla, CA, USA) was used. The *p*-value was calculated via Kruskal–Wallis test and Dunn’s multiple comparisons test. In the diagrams, box plots according to Tukey are shown. To evaluate the electrophysiological measurements, Microsoft Excel and Student’s *t*-test were used.

## 5. Conclusions

The results of our study demonstrate that the endothelial loss of WWC proteins causes a vascular hypersprouting in comparison to the control group. In addition, we identified WWC2 to be the crucial regulator of angiogenesis and WWC1 to be not decisively important in this context. Future studies will need to clarify whether remodelling can repair the vascular developmental malformations by inducing the knock-out postnatally and then investigate in adulthood.

## Data Availability

The data presented in this study are available on request from the corresponding author.
